# Landscape of intestinal microbiota in patients with IgA nephropathy, IgA vasculitis and Kawasaki disease

**DOI:** 10.3389/fcimb.2022.1061629

**Published:** 2022-12-16

**Authors:** Xueli Hu, Ru Fan, Wenzhu Song, Jianbo Qing, Xiaoyan Yan, Yaheng Li, Qi Duan, Yafeng Li

**Affiliations:** ^1^ School of Public Health, Shanxi Medical University, Taiyuan, Shanxi, China; ^2^ Department of Nephrology, Shanxi Provincial People’s Hospital (Fifth Hospital) of Shanxi Medical University, Taiyuan, China; ^3^ Core Laboratory, Shanxi Provincial People’s Hospital (Fifth Hospital) of Shanxi Medical University, Taiyuan, China; ^4^ Shanxi Provincial Key Laboratory of Kidney Disease, Taiyuan, China; ^5^ Academy of Microbial Ecology, Shanxi Medical University, Taiyuan, China

**Keywords:** IgA nephropathy, immunoglobulin A vasculitis, kawasaki disease, intestinal flora, commensal flora

## Abstract

**Objective:**

To explore the common differential flora of IgAN, Kawasaki disease and IgA vasculitis by screening and analyzing the differential intestinal flora between the three disease groups of IgAN, Kawasaki disease and IgA vasculitis and their healthy controls.

**Methods:**

Papers on 16srRNA sequencing-related intestinal flora of IgAN, Kawasaki disease and IgA vasculitis were searched in databases, the literature was systematically collated and analysed, the original data was download from the relevant databases, and then the operational taxonomic unit and species classification analysis were performed. Besides, Alpha diversity analysis and Beta diversity analysis were performed to screen for IgAN, Kawasaki disease and I1gA vasculitis groups and finally compare the common intestinal differential flora among the three groups.

**Results:**

Among the common differential flora screened, *Lachnospiracea_incertae_sedis* was lower in both the IgAN and Kawasaki disease groups than in the respective healthy controls; *Coprococcus* was low in the IgAN group but high in the IgA vasculitis group. *Fusicatenibacter* was lower in both the Kawasaki disease and IgA vasculitis groups than in their respective healthy controls, and *Intestinibacter* was low in the Kawasaki disease group, but its expression was high in the IgA vasculitis group.

**Conclusion:**

The dysbiosis of the intestinal flora in the three groups of patients with IgAN, Kawasaki disease and IgA vasculitis, its effect on the immunity of the organism and its role in the development of each disease group remain unclear, and the presence of their common differential flora may further provide new ideas for the association of the pathogenesis of the three diseases.

## Introduction

Immunoglobulin A (referred to as IgA) is divided into two types: serotype IgA and secretory IgA (SIgA), and secretory IgA is the main antibody in the local mucosal immunity of the body against infection (Pabst, 2012). Immunoglobulins have an important role in mucosal immunity, defense against pathogens that infect the mucosal barrier and development of immune tolerance, and in the respiratory and gastrointestinal tracts, the presence of immunoglobulin A in their mucosal tissues reflects its remarkable function (Pillai, 2013). Immune barrier dysfunction in the respiratory and gastrointestinal tract mucosa will be a risk factor for the development of various diseases. For example, IgA nephropathy is caused by IgA deposition in the kidneys; immunoglobulin A vasculitis (IgAV) is a systemic disease caused by IgA and complement component 3 (C3) deposition in small blood vessels; and there are related studies suggesting that Kawasaki disease may be a type of IgA vasculitis (Noval Rivas et al., 2019).

The pathogenesis of IgAN remains unclear, but one of the more established hypotheses suggests that galactose-deficient IgA1-IgG circulating immune complexes are deposited in the glomeruli through the bloodstream, causing renal lesions, often presenting with symptoms such as haematuria (Moldoveanu et al., 2007; Zhao et al., 2012). The development of KD is thought to be associated with abnormal activation of the body’s immune system, which produces high levels of immunoglobulins such as IgA and IgM (Kim et al., 2021). These globulins can form immune complexes with other substances and deposit them in small and medium-sized arteries throughout the body, causing an inflammatory response, particularly in the cardiovascular system, including the coronary arteries. IgAV, also known as allergic purpura, is a leukocyte-breaking vasculitis characterized by abnormal IgA deposition in the blood vessels, gastrointestinal tract and kidneys (López-Mejías et al., 2015) and is currently one of the most common primary glomerulonephritis worldwide (Guo et al., 2022). Elio G Carmona et al. identified a common genetic risk locus for KD and IgAV through a cross-phenotype meta-analysis (Sugiyama et al., 2020). Hitoshi Suzuki et al. strongly suggest that IgAN and IgA vasculitis with nephritis (IgA-VN) share similar signs and that galactose deficiency IgA1 plays a very important role in the pathogenesis of both (Suzuki et al., 2018; Suzuki and Novak, 2022). In conclusion, IgAN, IgAV and KD are all immune complex deposition diseases, but in addition, they may be closely related to genetic factors, environmental factors, immune function and other factors that affect the pathogenesis, but it is still unclear at present.

The mucosal immune system is an important part of the body’s immune network, consisting of the mucosal epithelium or mucosa-associated lymphoid tissue (MALT), which represents a physiological barrier and plays a very important role in the fighting against pathogenic micro-organisms, and is the body’s first line of defence against infection. The study of the mucosal immune system has provided ideas and methods for the study of the pathogenesis of many diseases. Previous studies have shown that the human intestinal microbiota is closely related to mucosal immunity (Macpherson and Harris, 2004). On the one hand, there are many immune tissues and cells in the intestine, especially in the ileocolic, such as diffuse lymphoid tissue, isolated lymph nodes, collective lymph nodes and T, B lymphocytes and macrophages (Frossard et al., 2015). On the other hand, the flora of the gut can influence the immune function of the gut by secreting mucus to fight off harmful bacteria and by regulating Treg cells through cytokines such as IL-17 (Rampoldi and Prinz, 2022). Once the flora is disturbed, the immune system will have recognition errors, leading to immune diseases in the human body. The development of IgAV is closely related to the dysregulation of the homeostasis of the gut microbiota. The abundance of gut microorganisms in children with IgAV is lower than in normal children (Li et al., 2022). In KD (Wang et al., 2018), children with the disease show a marked dysbiosis of the gut microbiota compared to healthy children (Khan et al., 2020). Genetic sequencing studies (Tang et al., 2022) have suggested that disruption of the intestinal mucosal barrier may be a key step in the pathogenesis of IgAN. This suggests that all three diseases are inextricably linked to the gut microbiota in some way.

This study aims to explore the similarities and differences in intestinal flora alterations in these three diseases which may be closely associated with IgA deposition, with the hope of suggesting new targets for disease prediction and contributing to the early prevention and treatment of the three diseases.

## Materials and methods

### Literature search

A computer search was conducted in Pubmed, Web of Science, Cochrane Library, CNKI, VIP and other databases using subject terms in combination with free words, and the search terms mainly included “IgA nephropathy”, “IgA vasculitis”, “Kawasaki disease”, “intestinal flora”, “intestinal microorganisms”, etc.; using “AND” and “OR” conjunctions to make a combination. The search period is from the creation of the database to 1 June 2022.

### Exclusion criteria for inclusion in the literature

Inclusion criteria: (1) ①IgAN in the literature is diagnosed by renal biopsy in patients with IgAN. ②Clinical signs and symptoms of IgAV patients meet the diagnostic criteria (Leung et al., 2020) for IgA vasculitis. ③The diagnosis of KD was established according to the guidelines of the American Heart Association (AHA) (McCrindle et al., 2017); (2) study type was a case-control study or cross-sectional study; (3) study technique: 16srRNA sequencing technology; (4) clear outcome indicators; (5) language limited to Chinese or English.

Exclusion criteria: (1) ①The diagnosis of IgAN in the literature was not confirmed by renal biopsy. ② Clinical signs and symptoms of IgAV patients do not meet the diagnostic criteria for IgA vasculitis. ③ The diagnosis of KD was not determined according to the American Heart Association (AHA) guidelines; (2) data not uploaded or not extractable; (3) duplicate publications; (4) literature with unclear subgroups and outcomes.

### Literature screening

Screening of the literature, evaluation of the quality of the literature and extraction and downloading of data from the included literature was done independently by 2 researchers in strict accordance with the inclusion and exclusion criteria of the literature, and in case of disagreement, the decision was made through discussion or consultation with a third party.

### Evaluation of the quality of literature

The quality of the literature was evaluated using the Newcastle-Ottawa Scale (NOS) (Cook and Reed, 2015) scale evaluation tool, which consists of eight modules, with higher scores indicating higher quality studies.

### Data extraction and downloading

The use of Excel sheets for the screened literature was collated. The SRA Toolkit tool in the NCBI Gene Database (https://www.ncbi.nlm.nih.gov/) was used to download the raw data associated with the literature that had been screened for 2 IgAN, 1 Kawasaki disease and 1 IgAV.

### Microbial information analysis process

The National Center for Biotechnology Information (NCBI) is the world’s leading bioinformatics resource, offering a wide range of access points to biomedical data and reliable analytical tools. Raw Data downloaded from the NCBI Gene Database (Raw Data) were quality controlled by merging, excising primers and splice sequences, removing chimeras, filtering, etc. (Edgar et al., 2011) Clustering and species classification analysis of the QC sample data was performed using USEARCH software (version 10.0.240) (Edgar, 2013). Operational taxonomic units (OTUs) were generated by clustering at a 97% similarity level. The OTU tables were then standardised. Species diversity was analysed using R software, including presentation of species abundance, alpha diversity and beta diversity. In beta diversity analysis, principal co-ordinates analysis (PCoA), one of the common methods used in Beta diversity analysis, is a method of dimensionality reduction of data based on a matrix of distances between samples and is used to analyse differences in microbial community composition between different ecological environments. The non-parametric rank sum test was used to detect differential colonisation between groups.

### Statistical analysis

R packages such as “ggpubr”, “ggplot2”, “patchwork”, “vegan”, and “cowplot” in R software were used to analyze the Alpha diversity and Beta diversity of the flora. The random forest classification using the R package randomForest model and for the selection of important flora. P<0.05 was considered statistically significant.

## Results

### Literature search results

**Figure 1:**
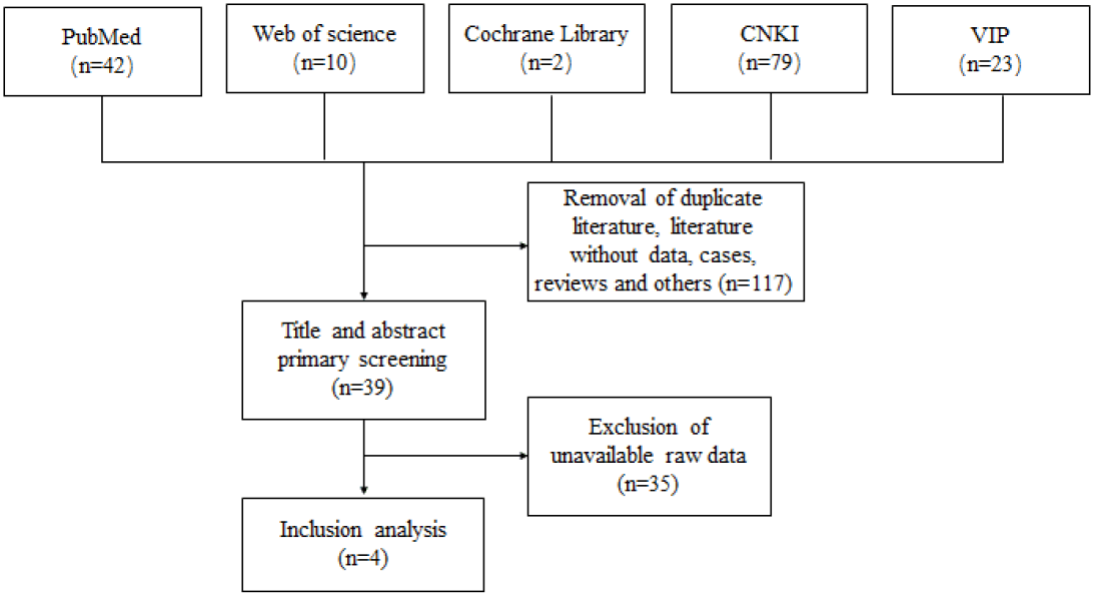
Flow chart for literature screening.

A total of 156 relevant publications were searched through Pubmed, Web of Science, Cochrane Library, CNKI and VIP databases. Among them, 42, 10, 2, 79 and 23 relevant papers were searched in Pubmed, Web of Science, Cochrane Library, CNKI, VIP and Wanfang, respectively. Duplicates, no data and other papers that did not meet the inclusion criteria were excluded, and a total of 4 papers were finally included in the analysis. The screening process could be available in **Figure 1**.

### Analysis of the relative abundance of species composition

**Figure 2:**
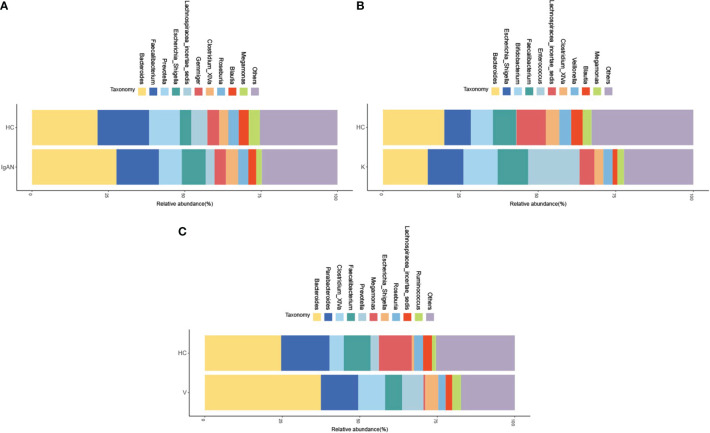
The ten groups of bacteria with the highest relative abundance at the level of the disease genera IgAN **(A)**, KD **(B)** and IgAV **(C)**.

In recent years, with the development of medical technology and research, the study of intestinal flora has gradually become a hot research content, and the study of intestinal flora has provided new ideas and new methods for the treatment of many diseases. Studies have found that the development of IgAN, Kawasaki disease, vasculitis and other diseases are all related to the dysbiosis of intestinal flora. In this study, it was found that at the genus level, the top ten groups of intestinal flora in relative abundance between the IgAN group and its healthy control (HC) group included *Bacteroides*, *Faecalibacterium*, *Prevotella*, *Escherichia_Shigella*, *Lachnospiracea_incertae_ sedis*, *Gemmiger*, *Clostridium_XlVa*, *Roseburia*, *Blautia*, *Megamonas*, and others. The relative abundance of *Prevotella*, *Lachnospiracea_incertae_sedis*, *Megamonas* in the IgAN group was significantly lower than in the HC group, but the relative abundance of *Escherichia_Shigella* was significantly higher than that of the HC group (**Figure 2A**). The top ten groups in the relative abundance of gut flora in the KD group and their healthy controls (HC) included *Bacteroides*, *Escherichia_Shigella*, *Bifidobacterium*, *Faecalibacterium*, *Enterococcus*, *Lachnospiracea_ incertae_sedis*, *Clostridium_XlVa*, *Veillonella*, *Blautia*, *Megamonas* and others. The relative abundance of *Bacteroides*, *Lachnospiracea_incertae_sedis*, and *Blautia* was significantly lower in the KD group compared to the HC group, but the relative abundance of *Escherichia_Shigella*, *Bifidobacterium*, and *Enterococcus* was significantly higher than that of the HC group (**Figure 2B**). The top ten groups of relative abundance of intestinal flora in the IgAV group and its healthy control group (HC) included *Bacteroides*, *Parabacteroides*, *Clostridium_XlVa*, *Faecalibacterium*, *Prevotella*, *Megamonas Escherichia_Shigella*, *Roseburia*, *Lachnospiracea_incertae_sedis*, *Ruminococcus* and others. The relative abundance of *Parabacteroides*, *Faecalibacterium*, and *Megamonas* were significantly lower in the IgAV group compared to the HC group, but the relative abundance of *Bacteroides*, *Clostridium_XlVa*, *Prevotella* and *Escherichia_Shigella* were significantly higher than those of the HC group (**Figure 2C**).

### Species-level taxonomical alpha diversity analysis

**Figure 3:**
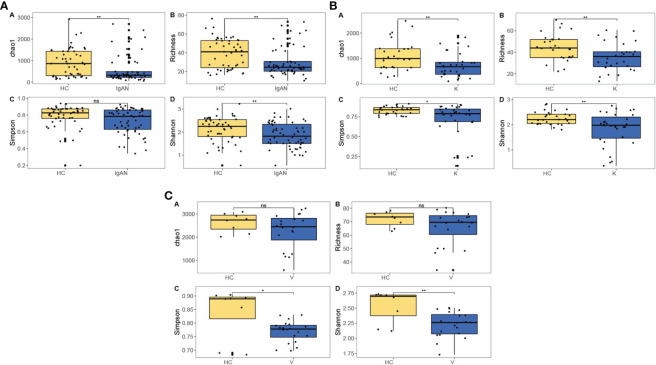
Intestinal flora richness and diversity in IgAN **(A)**, KD **(B)** and IgAV **(C)**. *P<0.05, **P<0.01, ns (no significance) P<1.

The results showed that at the genus level, the IgAN group had lower intestinal flora richness and diversity than the HC group (*P*<0.01) (**Figure 3A**); the KD group had lower intestinal flora richness and diversity than the HC group (*P*<0.01) (**Figure 3B**); and the IgAV group had lower intestinal flora diversity than the HC group (*P*<0.01) (**Figure 3C**).

### Species-level taxonomical beta diversity analysis

**Figure 4:**
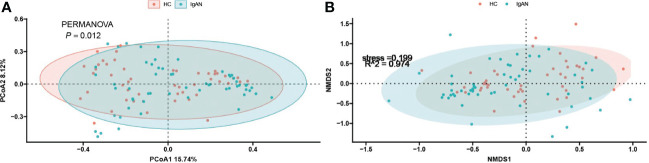
IgAN group gut flora PCoA analysis **(A)** and NMDS results **(B)**.

**Figure 5:**
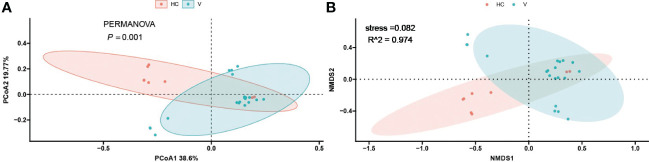
PCoA analysis **(A)** and NMDS results **(B)** for the Kawasaki disease group of intestinal flora.

**Figure 6:**
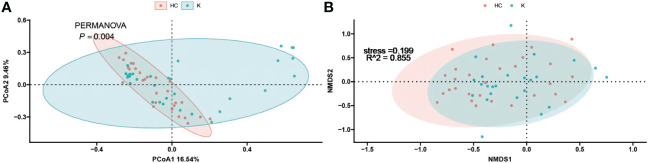
PCoA analysis **(A)** and NMDS results **(B)** for gut flora in the vasculitis group.

To investigate the differences in the composition of the gut microbiota between the disease and healthy control groups, the β-diversity between groups at the genus level in the disease and healthy control groups was analysed in descending order using principal coordinate analysis (PCoA) with Bray-Curtis distance and metric-free Multidimensional calibration (NMDS). In the IgAN group, PCoA1 was the principal component 1, explaining 15.74% of the overall flora. PCoA2 was the main component 2, explaining 8.12% of the overall flora (**Figure 4A**). NMDS analysis showed a stress value of 0.199, which was less than 0.2, and an R-squared of 0.974. This indicates that the results of this analysis are reliable and can show the degree of difference between the two groups (**Figure 4B**). In the KD group, PCoA1 explained 16.54% of the overall flora; PCoA2 explained 9.46% of the overall flora (**Figure 5A**). The NMDS analysis showed a stress value of 0.199, which was less than 0.2, and an R-squared of 0.855. The results of the NMDS analysis showed that the stress value was 0.199, which was less than 0.2, and the R-squared was 0.855 (**Figure 5B**), which was a more accurate response to the fact that the two groups of intestinal flora in this disease were different at the genus level. The IgAV group showed that the two groups of samples were far apart, indicating that the intestinal flora was significantly different at the genus level, with PCoA1 explaining 38.6% of the overall flora; PCoA2 explaining 19.77% of the overall flora (**Figure 6A**). The NMDS analysis showed that the stress value was 0.082, which was less than 0.2; the R2 was 0.974, which was a good fit with high reliability (**Figure 6B**). This could indicate a significant difference between the IgAV group and the HC group for this disease.

### Differential flora analysis

**Figure 7:**
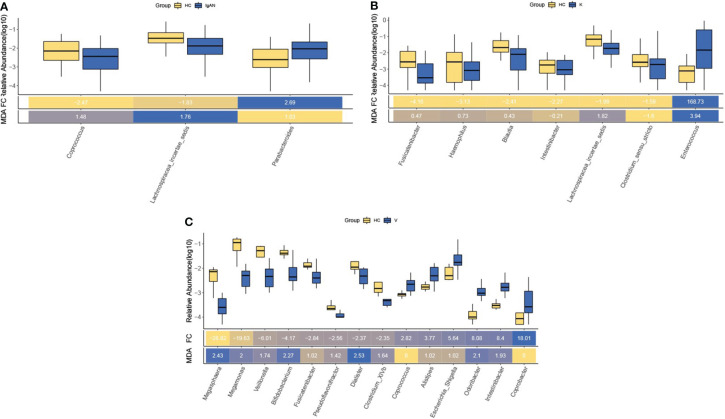
Differential flora analysis of the intestinal flora of IgAN **(A)**, KD **(B)** and IgAV **(C)**.

Analysis of differences between groups of intestinal flora in each disease group, screening for differential flora at *P*< 0.05, with MDA indicating the degree of reduction in the predictive accuracy of random forests, with larger values indicating greater importance for that flora. *Lachnospiracea_incertae_sedis* had the greatest importance among differential flora in the IgAN group versus healthy controls (**Figure 7A**). *Enterococcus* was of greatest importance in the differential flora of the KD group versus healthy controls (**Figure 7B**); *Dialister* was of greatest importance in the differential flora of the IgAV group versus healthy controls (**Figure 7C**). Among the differential flora screened for the three diseases, common differential flora was identified, with *Lachnospiracea_incertae_sedis* in the IgAN group versus the KD group; *Coprococcus* in the IgAN group versus the IgAV group; *Coprococcus* in the KD group versus the IgAV group *Fusicatenibacter* and *Intestinibacter*.

## Discussion

IgAN is a primary glomerulonephritis characterized by the deposition of immunoglobulin A in the glomerular mesangium. IgA deposition in IgAN is closely related to dysbiosis of the intestinal flora (Wang et al., 2022), which mediates the secretion of IgA in several ways, thereby contributing to the development of IgAN, including lipopolysaccharide, a product of dysbiosis of the intestinal microflora, which induces IgAN and stimulates the production of abnormal IgA1 by the Lymphocytes to produce abnormal IgA1 (Coppo, 2015). Interventions for gut microbial dysbiosis, such as fecal microbiota transplantation (FMT), where normal gut microbiota are introduced into the diseased host, will be a new therapeutic approach (Zhi et al., 2022). KD is an immune-mediated systemic vascular inflammatory disease and is closely related to immune dysregulation, which may be exacerbated by pathogenic infections (Yamashiro et al., 1996). It has also been found that KD, one of the immune disorders, is often associated with dysbiosis of the gut flora (Armaroli et al., 2019). IgAV is a common form of systemic vasculitis involving multiple organs in children. Recent studies have demonstrated that dysbiosis of the gut microbiota has an impact on the development of IgAV and that the abundance of actinomycetes may be related to the severity of the disease (Li et al., 2022).

It is generally accepted that the human gut flora consists of about 30 genera and 500 species of bacteria, the microbiota, and that these intestinal players work together to maintain homeostasis in the human gastrointestinal tract (Ling et al., 2022), ensuring the integrity of the epithelial barrier function of the gut and the normal function of various immune cells. Our group has found that dysbiosis of the intestinal flora is associated with many autoimmune diseases, such as primary Sjögren’s syndrome (Xin et al., 2022), systemic lupus erythematosus (Yaigoub et al., 2022) and Takayasu arteritis (TA) (Fan et al., 2022). But in this study, our analysis found significantly lower intestinal bacterial abundance and species richness in children with IgAV, KD and IgAN groups than in healthy children, but also a relatively increased number of genera in the different diseases. The relative abundance of Bacteroides, *Clostridium_XlVa*, *Prevotella*, and *Escherichia_Shigella* was significantly higher in the IgAV group than in the HC group, which is broadly similar to the findings of Jiaxing Tan et al. (2022), but they also suggested an increase in *Streptococcus* spp. and concluded that *Streptococcus* was positively correlated with the severity of the disease and the higher the increase in *Streptococcus*, the more pronounced the renal manifestations in patients with vasculitis. In the IgAN group, the relative abundance of *Escherichia_Shigella* was significantly higher than that in the HC group, which is consistent with the findings of Xiaofang Hu et al. who concluded that *Escherichia_Shigella* was negatively correlated with estimated glomerular filtration rate (eGFR) but positively correlated with urinary albumin to creatinine ratio (uACR), which has some significance for IgAN (Hu et al., 2020). In contrast, studies by Maria De Angelis and some national scholars have shown significant differences between *Bifidobacterium* in the IgAN group and healthy controls (De Angelis et al., 2014; Chen et al., 2020; Dong et al., 2020), but the increase in this genus in our study was more pronounced in KD. The relative abundance of *Escherichia_Shigella*, *Bifidobacterium*, and *Enterococcus* was significantly higher in the KD group than in the HC group, as corroborated by the study of Jie Chen et al. (2020). However, Imran Khan et al. proposed three bacterial pathogens that contribute to the pathogenesis of KD, namely *Fusobacteria*, *Shigella* and *Streptococcus (* Khan et al., 2020).

After random forest prediction analysis, we also identified the bacterial genus of greatest differential importance in the three diseases, *Lachnospiracea_incertae_sedis* in the IgAN group, *Enterococcus* in the KD group and *Dialister* in the IgAV group. Changes in these three bacteria in the early stages of the disease may indicate the onset of the disease. *Lachnospira* exists in the gut of most healthy individuals and may be a potentially beneficial bacterium involved in the metabolism of multiple carbohydrates. *Enterococcus* is a common conditional pathogenic bacterium in the intestinal tract. The onset of disease infection is associated with an increase in the abundance of this bacterium, creating a favourable environment for infection to occur, promoting the onset of infection and further participating in influencing the development of KD (Li et al., 2019). Intestinal colonization of *Dialister* has been shown to be a result of recognition and selection by the host immune system, which can interact through Toll-like receptors (TLR) and other specific microbe-host interactions. Members of this genus are capable of metabolizing glycans produced by the host as well as complex plant polysaccharides (Forbes et al., 2016). We also analysed the co-different bacteria between these three similar diseases. *Fusicatenibacter* is a type of anaerobic bacteria that enzymatically hydrolyzed undigested carbohydrates in the intestine and inhibits the inflammatory response by promoting the production of short-chain fatty acids, thereby controlling the secretion of inflammatory factors. In KD patients, the relative abundance of *Clostridium* spp. is low and cannot control the inflammatory response, further contributing to the development of the disease (Shan et al., 2018; Mu et al., 2020). Increased numbers of *Enterobacteriaceae* have also been found in many kidney-related diseases (Forbes et al., 2016). *Trichoderma* spp. also represents anaerobic bacteria and is often beneficial in the gut microbiota, metabolising short-chain fatty acids that protect the kidney and modulate inflammation (Wong et al., 2014). The relative abundance of *Lachnospiracea_incertae_sedis* was low in IgAN and KD patients. The reduction in the relative abundance of beneficial bacteria reduces their protective effect on the kidneys and their inability to modulate the inflammatory response, which may be associated with the development of disease. The co-different bacteria in IgAN and IgAV disease are *Coprococcus*, a genus of *anaerobic cocci*, an important genus of intestinal bacteria that ferment carbohydrates, and produce butyric and acetic acids and lactic acid. The relatively low abundance of *Coprococcus* in patients leads to relatively low production of butyric acid, allowing for disruption of intestinal barrier function, which may contribute to the development of the disease (Wu et al., 2022). In patients with IgAV, significant alterations in the intestinal flora of *Coprococcus* may indicate a preclinical stage of IgAN and vice versa. Similarly, *Lachnospiracea_incertae_sedis* in IgAN and KD, and *Fusicatenibacter* and *Intestinibacter* in KD and IgAV have the same suggestive effect. In summary, this paper suggests that *Lachnospiracea_incertae_sedis*, *Fusicatenibacter*, *Intestinibacter*, *Streptococcus*, and *Bifidobacterium* play a role in the pathogenesis of autoimmune diseases. However, their action mechanism and regulation styles require further studies for validation. In this study, our put the three diseases together and found out the co-differential flora that may affect the three immune diseases of IgAN, IgAV and KD or affect the specific flora of disease by comparison and analysis, but the mechanism of flora involvement in the disease may require further experiments for functional validation. In this study, raw data from four relevant papers were used for analysis, which is a relatively small amount of data. In the future, we will increase the number of samples from relevant patients for sequencing, analysis and validation to provide more adequate evidence for further studies and provide better ideas for exploring specific pathogenesis.

Overall, this study presents a new approach to disease prediction that may help in the early detection of IgAV, Kawasaki disease and IgAN, which may help in clinical prevention and treatment, delaying disease progression and changing the prognosis of the disease.

## Data availability statement

Publicly available datasets were analyzed in this study. This data can be found here: NCBI; PRJNA574226, PRJNA785415, PRJNA595748, and SAMN22814273.

## Author contributions

XLH drafted the manuscript. RF, WZS, JBQ helped polish the manuscript. XYY, QD, YHL gave precious advice on the analytical methods, and YFL was responsible for the conception and design of the research.
